# Translational medicine between human and veterinary emergency and critical care medicine: a story meant to have a happy ending

**DOI:** 10.1186/s13054-019-2659-3

**Published:** 2019-11-19

**Authors:** Nuno M. Félix, K. Gommeren, S. Boysen

**Affiliations:** 1 Departamento de Pediatria Hospital de Santarém, Departamento de Pediatria Hospital de Santa Maria, Centro Hospitalar Universitário Lisboa Norte, Lisbon, Portugal; 2 Faculty of Veterinary Medicine, University of Liège, Liège, Belgium; 3 Faculty of Veterinary Medicine, University of Calgary, Calgary, Canada

“Despite several fluid boluses and vasopressor support, mean arterial pressure hovered around 50 mmHg, lactate levels increased and ScvO_2_ remained at 55%. The patient was in septic shock. Should inotropic drugs be started? Physiologic steroids? Cardiac ultrasound …?”

To many emergency and critical care (ECC) physicians, this is a daily routine. Few imagine this patient being a dog or cat. However, small animal intensive care units (ICUs) exist. Human and veterinary emergency and critical care (HECC and VECC) share attitudes and values, providing opportunities for the development of both professions.

Advancements in ECC rely on animal experiments [1, 2], particularly rodent models due to large litter sizes, short generation time, relatively low maintenance requirements, and the ease of introducing genetic modification in these species [2]. These characteristics make translational medicine from rodent-based models to humans fundamental to understanding the pathologic mechanisms behind many human ECC conditions [3].

However, the translation of rodent-based research to clinical practice has been questioned for several reasons, including the use of young, single-sex, genetically modified, healthy (or limited comorbidities), inbred subjects, kept in homogeneous, regulated environments [1–3]. Recent studies have also suggested that rodents and humans may differ more in their genetic response to acute injury than originally believed [2, 3].

Sheep, pigs, rabbits, and non-human primates have also been used in ECC research [4]. Proposed advantages of these species include their larger size, allowing advanced monitoring similar to what exists in human ICUs, ability to create predefined comorbidities, and the fact these species have similar hemodynamic and cardiovascular responses to humans. However, large animal models are relatively expensive, have limited immunologic markers available in some species (e.g., sheep), and share similar limitations to rodent models (e.g., single-sex experiments) [4].

Although valuable in preclinical research, a gap still exists between animal models and the clinical human ECC setting [1–3, 5]. In our opinion, this gap can be partially filled by studying companion animals [5] as a model for ECC human diseases, for several reasons.

First, most HECC scenarios (e.g., sepsis) also exist in companion animals, with similar pathophysiologic mechanisms. The similarity is especially remarkable in pediatric patients, which are less afflicted by lifestyle choices like smoking and alcoholism (not observed in companion animals) and complications of chronic diseases (e.g., diabetic nephropathy). Dogs and cats, having shorter life spans, rarely manifest such complications.

Second, companion animals have complex genetic, environmental, and physiological variation, which in many cases are similar or even shared with humans [5, 6].

Third, current ECC management of critically ill dogs and cats is similar to humans. Veterinarians have access to advanced diagnostics including molecular testing and advanced imaging. In VECC, capnography, invasive hemodynamic, and ICP monitoring are performed, and sidestream dark-field imaging and tissue oxygen saturation have been used clinically. Advanced therapeutic modalities in VECC include ventilation, interventional radiology, advanced hemodynamic support, transfusion therapy, continuous renal replacement therapy, and cardiac bypass, among others.

Fourth, current challenges faced by VECC are similar to those in HECC: specific markers of sepsis, synthetic colloids in septic patients, identifying volume-responsive patients, mechanical ventilation strategies, antibiotic selection and treatment duration, and transfusion strategies in trauma, just to name a few.

Therefore, we believe cooperation between HECC and VECC can provide a bidirectional, synergistic pathway, with benefits for both by the following:

- *Improving the knowledge of critical disease pathophysiology.* This has been reported for spinal cord injury [7], epilepsy [8], cardiomyopathies [9], asthma [10], and cancer [11, 12]. In oncology, the Comparative Oncology Genomics Consortium, within the National Cancer Institute and the Purdue Comparative Oncology Program (PCOP) [11], have discovered new aspects of carcinogenesis and cancer biology and facilitated the development of new diagnostics and therapeutics [11, 12].
- *Bridging preclinical data and human clinical trials, to evaluate safety and efficacy of new treatments.* This is one of the strongest arguments to support HECC and VECC collaboration. An example was the canine trial of ibrutinib in naturally occurring B cell malignancy that accelerated its FDA approval for human mantle cell lymphoma [12].
- *Providing new therapeutic alternatives.* Cyclosporine-based ophthalmic emulsion for the treatment of keratoconjunctivitis sicca was first reported by a veterinary ophthalmologist [13].

## Limitations

Limitations for translating VECC-generated data to HECC do exist. Genetic, anatomical, and physiological differences between species remain a limitation of companion animal models. For example, the highly contractile nature of the canine spleen, which can store and auto-transfuse a larger blood volume than the human spleen, plays a greater role in the response to hemorrhage than humans [4]. In addition, dogs and cats may be less applicable than sheep or pigs when size-related variables are important research considerations. Additionally, because costs are primarily owner driven in VECC (pet insurance is not readily available worldwide), economic factors play a significant role in medical decision-making. Moreover, euthanasia is ethically acceptable for animals in specific circumstances. These factors may limit the intensity and/or duration of ECC treatment in VECC, although this will more heavily impact the translation of HECC data to VECC than the reverse.

## Concluding remarks

Board-certified specialization in VECC exists through the American and European Colleges of Veterinary Emergency and Critical Care (ACVECC and ECVECC). Residency programs are available in many parts of the world, and international collaborations are becoming a commonplace. Consensus guidelines regarding several subjects are now available (e.g., cardiopulmonary resuscitation) [14] or in development.

Interestingly, the collaboration between HECC and VECC has already begun. During the 2010 Multi-Disciplinary Review/Post-Graduate Review in San Diego, human trauma surgeons, VECC specialists, and translational medicine scientists discussed the use of naturally occurring canine trauma as a model to human trauma (Spontaneous Trauma in Animals Team). This led the ACVECC Veterinary Committee on Trauma to create a clinical research infrastructure to enhance uniform delivery of care and permit large-scale clinical trials in canine and feline trauma patients [15].

In conclusion, we believe the use of companion animals to model human ECC can complement current available preclinical animal models, with benefits to all species. To achieve this, VECC and HECC should increase and promote the collaboration which already exists. We just have to say yes and seize the opportunity.

## Data Availability

Not applicable
